# Supplementary material for: Municipal officials’ subjective distress in coordinating with the national government during the decontamination project of radioactive materials in Fukushima: A qualitative study
Source: PLoS One. 2026 Apr 3;21(4):e0346597. doi: 10.1371/journal.pone.0346597 (PMC13048426; doi:10.1371/journal.pone.0346597)
Supplement: Attachment — Submitted filename: PONE-D-25-47633v1.pdf [file pone.0346597.s003.pdf]

1. General comments

   The reviewer commends the authors' efforts to enhance the quality of research papers aimed at reducing the psychological burden on local government officials. High-quality qualitative research, unlike many quantitative studies, will bring immeasurable insights to humanity.
   There may be value in achieving catharsis by addressing dissatisfaction with national government officials, which local government employees find easier to voice. However, from the perspective of solving fundamental issues, improving the relationship between local government officials and national government officials is essential. Without such efforts, as the authors themselves acknowledge, it risks exacerbating divisions. Of course, a single paper cannot resolve all issues concerning overly complex challenges as authors explained, but isn't the framing of the problem important? This is an unfair hypothetical question, and authors would be wise to ignore such abusive behavior from the reviewer. However, the reviewer became curious about what insights might be gained if these authors were to establish connections with national government officials and conduct a similar investigation. Given that the authors have built relationships with research collaborators over many years, they should highlight the benefits this has brought to local government workers.
   Authors may share this reviewer's comments with the local government.

2. Title
   - ~~A) If authors believe there are limitations to generalization, why not explicitly state that it is a case study?~~
   - ~~B) It states that “radiation decontamination,” but what was decontaminated was not radiation but radioactive material; the term “radiation decontamination” is not rationale.~~

3. Abstract
   - ~~A) Line 35: “may be misaligned with local needs;”~~
     ~~This is an ambiguous expression. As it lies at the core of the issue raised in this paper, it is essential to present the factual basis within the main text. Otherwise, it cannot be considered a scientific paper.~~
   - ~~B) Line 39: “limited human and financial resources”~~

~~The Japanese government's decontamination costs amount to over 12 billion Euro with local governments outside Fukushima also providing personnel support (which has had various impacts on dispatched staff). While it may not be appropriate to directly compare each situation, discussions must take into account how local governments have responded to other environmental contamination incidents both domestically and internationally.~~

4. Introduction
   A) Line 49: “play key roles”
      The reviewer believes that the burden on city employees regarding decontamination projects stems from their relationship with residents, but t reviewer respects the authors' choice not to agree with this (primary group of interest in an academic paper should be focused). However, the reviewer has doubts about the social nature of the argument presented in this paper (whether it conveniently focuses only on easily discussable elements).
   B) Line 60: “mandated by the national government”
      The reviewer respects the authors' judgment that discussing this point is redundant, but considers it factually incorrect in the field of public administration from a legal perspective as well. Note that the reviewer does not state that this discussion should be conducted.
   C) Line 65: “little is known”
      The validity of the analytical approach itself, which focuses specifically on the perspective of relationships with national government officials, is being questioned. Regarding the examination of the burden on local government employees, data on resignations and leaves of absence among young employees in each municipality may also be available.
   D) Line 77: “These characteristics may be the reasons for the reluctance of the affected MGOs to disclose their views regarding the national government”
      Is the authors' inference that local governments would not have agreed to the survey otherwise shared with the municipalities? Were deceptive techniques used in requesting cooperation for the survey from local governments?

E) Line 79: “their anonymity must be protected;”
The reviewer emphasizes the importance of respecting the autonomy of MGOs who contribute to the research. However, do the authors' concerns that this approach could potentially cause problems align with the perspectives of the MGOs themselves?

~~F) Line 85: “in a difficult relationship with the national government.” What are the administrative science-based reasons for the difficulty?~~

~~G) Line 88: “potential countermeasures” Given the amount of time that has passed, authors should be able to make proposals based on scientific evidence that incorporates all previous attempts.~~

H) Line 89: “hypothetical model”
Readers are interested in general hypotheses and how those hypotheses/research questions were verified. Can the insights gained be applied to improve operations on the ground?

5. Methods

A) Line 104
How did authors explain the necessity of indicating gender?

B) Line 243: “Line 104”
Is the authors' judgment that this study does not constitute “insufficient recruitment” based on the opinion that further expansion of the investigation is unnecessary?

6. Results

~~A) Line 260: “illustrated the divergence in priorities between the national government and local stakeholders.” What differences were there in terms of priority? And how did those differences affect the “ultimate decontamination goals”?~~

~~B) Line 271: While coexisting with the temporary storage area involved various ingenious solutions, what function did the interviewer's responses to rhetorical questions aim to serve, considering these efforts? Can anyone other than Japanese people even grasp the significance of this rhetorical question in the first place?~~

~~Does this indicate the authors' interpretation that it has become a convenient excuse to avoid confronting the underlying difficult issues?~~

- C) ~~Line 272:~~

  ~~Are authors assuming that readers would think, "It goes without saying that just because the scheduled period has ended doesn't mean the work is finished"?~~

- D) ~~Line 290:~~

  ~~Considering the context from the previous sentence, do you think this passage is pointing out that they don't understand the know-how regarding what?~~

- E) ~~Line 293:~~

  ~~Is this pointing out a lack of opportunities to communicate with suitable personnel, or is it highlighting a different issue? If it's the latter, what kind of improvement ideas might be considered? What elements do the authors speculate this uncertainty pertains to? Regarding the transition in the final sentence here, is the reader expected to recognize that the logic doesn't hold?~~

- F) ~~Line 310:~~

  ~~Additional information regarding support staff from other municipalities would also be helpful.~~

- G) ~~Line 323:~~

  ~~At that time, this was a concern frequently voiced by residents, but it would also be helpful to add how businesses and other establishments responded to it.~~

- H) ~~Line 340:~~

  ~~If the information provided by the Ministry of the Environment and local governments was insufficient to counter the spread of misinformation, it would be better to explain why that was the case.~~

  ~~https://www.env.go.jp/en/~~

7. Discussions

   - A) Line 541:

     The authors' proposed improvements represent "a realistic and contextually grounded alternative," which I understand to mean that the authors recognize the national government's approach is

not “a realistic and contextually grounded alternative.” Therefore, the reviewer's suggestion here is that explicitly stating what aspects of the national government's approach are not “a realistic and contextually grounded alternative” could help facilitate improvements at the local level.

8. Conclusions
   - ~~A) Line 19, Page 16: “The of ages of artifacts…”~~
     ~~The first “of” would be unnecessary.~~
   - ~~B) In the conclusion, authors should concisely state what this study has revealed. From this perspective, the introduction must state what research hypothesis this study aims to clarify and why verifying that hypothesis is important.~~

8. Data availability statement

~~While it is considered to be anonymized, it would be beneficial to express opinions on the disclosure of speech records from the perspective of open science.~~

9. Table 2
   - ~~A) “national radiation exposure standards”~~
     ~~Considering how it aligns with IAEA GSR Part 3, it would be better to explain why municipal government officials felt dissatisfied.~~
   - ~~B) “through staged site visits by politicians”~~
     ~~It would be better to explain what intricate schemes were involved, taking into account the disclosure of facts regarding the bureaucrats' briefing to politicians. In particular, it would be better to clearly show how the mayors and the prefectural governor countered such treatment.~~
   - ~~C) It would be good to highlight what protests Fukushima Prefectural Assembly members and members of local assemblies within Fukushima Prefecture carried out, and how Japanese citizens outside Fukushima Prefecture ignored those protests.~~
   - ~~D) “fluid national policy”~~
     ~~It would be helpful to include supplementary information on the mechanisms by which “fluid national policy” was formed.~~
     ~~It would be good to also highlight the challenges of flexibly revising plans as the situation evolves.~~

~~E) “The plan lacks a perspective on decontamination for the sake of residents' lives when they returned”~~

~~The explanation of how this situation came about is also important. In this explanation, the lawsuit filed by Tsushima in Namie Town, Futaba District, can also be cited as an example (the plaintiffs' group's website—which was inaccessible for a long time, yet the plaintiffs endured this—contains a significant error in the English translation of the plaintiffs' group's name, which remains uncorrected).~~

~~F) “frustration at the snail's pace of decontamination”~~

~~If he was explaining a delay in the plan, it is helpful to supplement authors’ explanation with why the delay occurred and how much time was lost.~~

~~G) “decontamination by the national government's initiative as just for show.”~~

~~It would be helpful to explain the power balance structure that prevented the municipal government from taking the lead.~~

~~H) “the never-ending process”~~

~~It would be helpful to explain what process is being referred to and why it is endless.~~

~~I) “The dishonest attitude of the national government towards residents”~~

~~It would be helpful to have specific examples.~~

~~J) “the lack of conviction in national government explanations”~~

~~It would be good to indicate what kind of “conviction” is expected.~~

~~K) “These risks exacerbate by the limitations of 'top management' without the knowledge of the local situation, leading to wrong decisions.”~~

~~It would be better to clarify what ‘top management’ refers to.~~

~~L) “looking down”~~

~~It would be better to clarify what ‘looking down’ refers to.~~

~~M) “a lack of respect for our expertise and efforts”~~

~~It would be better to clarify what 'expertise and efforts' refers to.~~

~~N) “not keeping records”~~

~~It would be better to clarify what ' not keeping records' refers to.~~

~~O) “keep disaster records”~~

It would be helpful to explain why the difficulty of this administrative work cannot be overcome through the use of ICT.

P) “by corner-cutting by national government officials on record”
Here, a municipal official was criticizing national government officials for failing to keep records, was not it? By automatically recording the national government officials' failure to keep records, could this effectively take over the task of record-keeping from the national government officials?

Q) “promises that are not kept because of changes in personnel”
It would be beneficial to also present the Japanese government's response to the written question submitted by the Fukushima-elected Diet member, which demanded an explanation for why the promises were not kept, after presenting the official documents detailing what was agreed upon between national government officials and municipal government officials.

R) “Their approach, including the use of compensation money used to suppress opinions, does not address the root issues.”
Compensation money is not intended for decontamination efforts. It would be helpful to explain what ‘suppress’ refers to.

S) “the ideas from frontline feedback”
It would be better to clarify what ' the ideas from frontline feedback ' refers to.

T) “the repeated betrayal by the national government”
It would also be beneficial to show how government agencies conducted briefings for Diet members elected from Fukushima Prefecture, as this has already been attempted by Fukushima residents.

U) “limited autonomy.”
If the inherent “autonomy” is impaired, it would be helpful to have an explanation as to why.

V) “excessive responsibility to plan and implement the timing of the lifting of the evacuation”
It would be helpful to have an explanation of what constitutes ‘excessive’ responsibility for matters that should fall under the jurisdiction of municipal government officials.

W) “conflicting interests and priorities”

~~It would be better to explain who is in conflict with whom. FMU experts are also contributing to resolving the conflict taking into account the positive precedents set overseas, aren't they?~~

~~X) "The reduced trust damages the relationship between municipal government and residents, and makes secretive attitude of local residents leaders"~~

~~The attitude of local residents' leaders is a crucial factor in addressing the issue, and it would be helpful to clarify what "secretive" refers to here. Additionally, an explanation of "local residents' leaders" would be beneficial.~~

~~Y) "the most suitable site for waste disposal and temporary storage is not selected."~~

~~Given that this location is optimal and does not compromise fairness, it would be beneficial to supplement this by explaining what issues existed in the decision-making process.~~

~~Z) "Compounding these challenges is the money-grabbing behaviour by residents."~~

~~It would be helpful to have supplementary information on the relationship between this assignment and Stage 1.~~
