# Supplementary material for: Municipal officials’ subjective distress in coordinating with the national government during the decontamination project of radioactive materials in Fukushima: A qualitative study
Source: PLoS One. 2026 Apr 3;21(4):e0346597. doi: 10.1371/journal.pone.0346597 (PMC13048426; doi:10.1371/journal.pone.0346597)
Supplement: Attachment — Submitted filename: response.docx [file pone.0346597.s004.docx]

We would like to express our sincere gratitude for the careful and insightful reviews. The reviewers’ expert comments have been extremely helpful in improving the manuscript.

Before providing our point-to-point responses to each comment, we would like to outline three major revisions made in the revised manuscript:

1) In accordance with the editor’s instructions, we revised the manuscript to ensure compliance with the appropriate formatting requirements.

2) Following Reviewer #1’s concerns regarding the SCAT analytic procedures, we fundamentally reconsidered the analysis; as a result, the previously presented five-stage model has been reorganised into four stages.

3) In response to Reviewer #2’s observation that the term “radiation decontamination” was unnatural, we revised the title accordingly.

All revisions in the manuscript are highlighted in yellow. As these adjustments entailed substantial changes to the paper’s overall content—especially the Results section, which has been entirely rewritten, along with the corresponding updates to the Discussion—we recognise that this imposes an additional burden on the reviewers during re-evaluation. We kindly ask for your understanding that these extensive revisions were necessary to address the reviewers’ comments adequately.

[Editor]

Q1. Please ensure that your manuscript meets PLOS ONE's style requirements, including those for file naming. The PLOS ONE style templates can be found at…

A1. Thank you for your comment. We have read the guidelines and revised our manuscript. If any further aspects require revision, we would appreciate your guidance.

Q2. In the online submission form...All PLOS journals now require all data underlying the findings described in their manuscript to be freely available to other researchers, either 1. In a public repository, 2. Within the manuscript itself, or 3. Uploaded as supplementary information...

A2. The interview scripts were included as a supplementary file with all personally identifiable information removed. Therefore, the present case corresponds to option 3 “Uploaded as supplementary information”.

Q3. Please include captions for your Supporting Information files at the end of your manuscript, and update any in-text citations to match accordingly. Please see our Supporting Information guidelines for more information: http://journals.plos.org/plosone/s/supporting-information.

A3. We have added the caption to the supporting information (in manuscript p.37)

Q4. We note that this data set consists of interview transcripts. Can you please confirm that all participants gave consent for interview transcript to be published?...

A4. The interviewees provided consent for the interview transcripts to be published. However, the previous manuscript included specific dates that could potentially lead to personal identification. To ensure anonymisation, we have revised the description of the interview schedule in the Methods section as follows: ‘We conducted one interview with Mr X and Mr Y in 2015, and five interviews with Mr Z between 2016 and 2017.’ (p. 7)

[Reviewer#1]

Q1. There is no description of the analysis procedure, including what SCAT is. The manuscript does not explain how the authors analyzed the data and derived their results. The Methods section should specify what the segments (theoretical segments), components, and themes in the results represent, and how they were generated. In addition, is the term "theoretical segment" appropriate? In SCAT, a “segment” refers to a piece of data entered into a single row of the analysis spreadsheet (Otani T. Paradigm and Design of Qualitative Study: From Research Methodology to SCAT. The University of Nagoya Press, 2019, p. 280).

A1. We appreciate the reviewer’s comment. The term ‘segment’ was indeed a mistranslation and therefore inappropriate; we have removed it and replaced it with more suitable expressions throughout the manuscript—for example, ‘narrative excerpts’ instead of ‘speech segments’, and ‘theory from Mr X/Y/Z’ instead of ‘theoretical segment’.

Regarding SCAT, we acknowledge that the explanation in the previous version, although present in the Qualitative Analysis subsection of the Methods section, was insufficient. We have therefore added the following information to clarify our analytical procedure (pp. 8-9):

“The verbatim transcripts of all the interviews were analysed cross-sectionally using Steps for Coding and Theorisation (SCAT), a qualitative data analysis method for inductively summarizing narratives, as described in previous studies [19-21]. SCAT has the advantage of extracting important excerpts from the original narrative and gradually abstracting them, ultimately deriving concepts while considering the meanings embedded in the original narrative. Specifically, we used the following seven steps: (1) extracting narratives that were considered important and rendering them into text; (2) selecting noteworthy words or phrases from the text (Step 1) to set the focus of the analysis; (3) paraphrasing the words and phrases extracted in Step 2 to convert the interviewee’s unique expressions into analyzable and interpretable forms; (4) identifying underlying regularities such as cause-and-effect relationships, sequential patterns, and/or tendencies within the data by using concepts from academic or professional domains that account for paraphrased texts in Step 3; (5) describing the core meanings expressed in the original narratives in abstract terms as concise headings—composed of nouns or noun phrases and referred to as “themes”—based on the results of Steps 2–4, in a way that generates novel conceptual constructs; (6) constructing a storyline that explains the interviewee’s experiences by using all themes generated in Step 5, supplementing them with additional wording where necessary; (7) by articulating the storyline in a generalized and unified descriptive form, describing the "theory" which is the brief and declarative expression what can be claimed based on the analysis of the text. The theory generated in Step 7 is the outcome of SCAT. Note that SCAT analysis is, in principle, conducted for each interviewee; accordingly, in this study as well, three distinct theories, corresponding to the number of interviewees, were ultimately generated.”

In accordance with SCAT’s formal procedures, we have also revised our analytical steps. Therefore, as noted above, please be aware that a separate theory was generated for each interviewee.

Q2. What are the four sentences (theoretical segments) in Table 2? They resemble “storylines” in SCAT. However, the purpose of the analysis is to generate theory, and the results should be theoretical rather than storylines (Otani T. Paradigm and Design of Qualitative Study: From Research Methodology to SCAT. The University of Nagoya Press, 2019, p. 311).

A2. Thank you for your comment. The analytic procedures were inappropriate. As the reviewer correctly points out, in SCAT, ‘theory’ refers to a written account developed from the storyline, characterised by generality, predictability, and coherence (source: p. 324 of Otani’s work cited by the reviewer). As described below, we have re-analysed the data and, accordingly, both the content and the presentation of the resulting theories have been substantially revised.

The number of theories ultimately generated is now reported in the Qualitative Analysis subsection of the Methods section (p.9):

“...as a result, 38 descriptions of theories were identified: 12 from Mr X, 8 from Mr Y, and 18 from Mr Z. These theories and underlying themes and storylines...”

In addition, the theories—conceptually distinct from the storyline—are explicitly presented in Table 2 as the results of our analysis. These are reported separately for each interviewee (Table 2, pp. 39–42). With these revisions, the content of the previous Table 2 has been replaced with an updated version.

Q3. Another concern is that a single theoretical segment (which appears to be a storyline) contains themes derived from multiple participants. In SCAT, each interview preserves its own contextual specificity, and therefore three interviews should yield three individual analytic results. While it is acceptable to later discuss the findings from four perspectives, the analysis itself should first be conducted for each participant individually, and only at the discussion stage should the results be reorganized into four perspectives (Otani T. Paradigm and Design of Qualitative Study: From Research Methodology to SCAT. The University of Nagoya Press, 2019, p. 367).

A3. The reviewer’s concern is valid. In accordance with SCAT’s formal procedures, we have re-conducted our analysis. We first generated a theory for each interviewee and reported these in the Results section. We then reorganised the theories derived from each interviewee to construct a model inferred from these analytic outcomes. Revisions have been made to document these analytical and interpretive procedures clearly.

First, we have added the following paragraph at the end of the Qualitative Analysis subsection in the Methods section, describing the creation of the model through reorganisation of the results (lines 210-217, p. 10):

“In the original SCAT procedure, the analysis is conducted and reported for each interviewee. However, because this study aimed to identify overarching patterns across participants, the researcher conducted an additional interpretive integration process that is not part of the standard SCAT procedure. Specifically, the individual theories generated through SCAT were further examined, compared, and conceptually grouped into higher-order categories through interpretive abstraction. This supplementary step was conducted in the discussion section to enhance integrative understanding while maintaining fidelity to the original SCAT principles by developing a hypothetical model of the process by which MGOs’ subjective distress becomes manifest.”

Second, because a separate theory was generated for each interviewee, we have added a new subsection to the Discussion section titled ‘National–municipal government relationship and MGOs’ hardship: individual-level findings’ (p. 20), in order to discuss the findings derived from each interviewee’s theory. We have also provided detailed explanations in this subsection (lines 448–456, 464–468, 487–498, and 520–523).

Third, we have provided a detailed explanation of the model obtained through the reorganisation of the theories in the subsection ‘Hypothetical model of MGOs’ subjective distress: Reorganising theories’ (p. 23). These represent the study’s integrated findings, and their four-step structure is summarised in Table 3. In the main text, new explanations have been added in lines 531–543, 545–547, 558–560, 562–569, 571–573, 578–584, 586–592, 606–607, and 620–627.

Please note that we have clearly distinguished between the theory generated by the standard SCAT procedure and the model derived from our interpretive (conceptual) analysis.

4. The Introduction should include at least a minimal explanation of the decontamination activities conducted in Fukushima.

A4. Thank you for your comment. We have added the explanation to the first paragraph of the Introduction section (lines 50-54, p.3):

“The decontamination project was a large-scale program aimed at removing radioactive materials released into the environment following the FDNPP accident, and it was an essential process for enabling residents to return and for promoting regional reconstruction[3]. Under the government's leadership, municipalities affected by contamination were legally obligated to engage in decontamination work[3]. Therefore...”

[Reviewer#2]

Before reporting the specific revisions made to the manuscript, please allow us to offer an overall, dialogic response to your comments.

Comment 1:

This study presents five perspectives, focusing particularly on the complexity of the relationship between municipal governmental officials and the national government not mentioning contributions from Fukushima Prefectural government officials. However, these “relationship challenges” tend to be easily expressed in society. Consequently, it may simply be that easily articulated grievances are surfacing. Furthermore, the paper does not present the underlying structural social context and administrative science perspective for the specific challenges discussed. Presenting analysis results without deeper exploration risks merely deepening divisions and hindering constructive progress. While resource constraints are emphasized, decontamination costs amount to approximately 4 trillion yen, representing a significant scale of response costs for an accident-causing environmental pollution compared to other cases. However, such comments may be perceived by the authors—who are psychologically attached to the research subjects—as an unethical attitude that fails to understand the suffering of the affected municipalities, as evidenced by the recorded testimonies. Therefore, it would be better to logically present the structure that inevitably leads local government employees to harbor dissatisfaction with the national government. Furthermore, while individualized responses tailored to personal psychological states may be effective in specific situations, this issue is fundamentally societal in nature, raising concerns that this perspective may be being overlooked.

Answer 1:

The reviewer’s comments are critical. First, regarding the perspective on “relationship challenges,” we agree that this should be explicitly acknowledged as a limitation of the study. Accordingly, we have added the following statement to the Limitation section (lines 653–661, pp. 28–29):

“Fourth, it should be acknowledged that the difficulties disclosed by the interviewees—such as their subjective distress or their strained relationships with the national government—represent only part of MGOs’ experiences and may not capture them in their entirety. It is possible that interviewees found it easier to speak about their difficult relationships with the government; if so, we must consider the possibility that some information remains undisclosed. There is an appropriate time to elicit and understand MGOs’ experiences. In this study, interviewees were able to share their stories because a long period had passed since the disaster, and conditions were in place to ensure that no individuals could be identified. However, building a longer-term relationship of trust between researchers and participants may create further opportunities for a richer and more in-depth qualitative inquiry.”

Second, the reviewer’s concern about the insufficient incorporation of structural social context and administrative science perspectives is well-founded. However, it is practically impossible for a single paper to comprehensively cover the diverse expertise involved in psychological, structural–sociological, and administrative science perspectives—and, if extended further, potentially political science, organisational science, and behavioural science as well. Importantly, although we are particularly interested in the psychological aspects of MGOs and in providing support for them, this does not mean we underestimate the value of structural, social-contextual, or administrative science approaches.

At the same time, our primary aim in this study was to analyse MGOs’ experiences from the standpoint of occupational mental health and psychological support, and to contribute findings that have practical relevance for frontline practice. We view the structural, social-contextual, and administrative science perspectives, as well as the psychological and occupational health perspectives, not as competing but as mutually important. Nevertheless, there are inherent limitations to accommodating all of these domains within a single manuscript.

In light of these considerations, and in order to clarify our position, we have added the following statements to the manuscript (lines 578–584, p. 25; lines 640–644, p. 28; lines 689–692, p. 30):

“While such individual-level mental health interventions are indispensable for supporting MGOs, it is important to recognise the risk that organisations may overly attribute the dilemma to “individual adaptability,” thereby overlooking conflicts rooted in structural deficiencies or flawed policy design. In parallel with mental health support, additional measures aimed at preventing MGOs from falling into such dilemma states—such as refining communication techniques to enhance the quality of dialogue between national officials and residents, or deploying mediators other than MGOs—may contribute more fundamentally to preventing these problems.”

“It should be particularly emphasised that strengthening both macro-level measures—grounded in institutional design and organisational science—and micro-level, interpersonal interventions—such as occupational mental health and psychological support—will enhance societal resilience to future disasters and safeguard MGOs as workers.”

“Consequently, we are convinced that strengthening both macro-level measures—grounded in institutional design and organisational science—and micro-level interventions—such as occupational mental health and psychological support—is essential for reducing subjective distress among MGOs and advancing post-disaster reconstruction efforts.”

Addressing the reviewer’s concern regarding the risk of “merely deepening divisions and hindering constructive progress” is indeed essential, and we regard this as the authors’ responsibility. At the same time, opportunities for MGOs to articulate their experiences are limited; in general, they tend to be reticent. It is nevertheless true that society has much to learn from MGOs’ narratives, and that these insights should be utilised in future recovery efforts. For this reason, by explicitly incorporating the reservations and explanations described above into the manuscript, we intended to report on MGOs’ circumstances while avoiding social polarisation and, at the same time, to lay a foundation for future discussion. We wish to emphasise that the findings of this study remain open to future research grounded in structural social-contextual and administrative science perspectives.

Third, we consider that the structural mechanisms and specific processes through which MGOs come to feel dissatisfaction toward the central government were presented through the narratives analysed in this study. At the same time, the reviewer is correct in noting that individual-level responses have clear limitations in addressing such “structural” issues. We also recognise that the effectiveness of individual interventions is limited and that improvements are required from broader societal and structural perspectives. To balance the fundamentally social nature of this issue with the need to propose feasible psychological and mental health support measures, we included statements acknowledging the importance of societal considerations, as described in our second response above (lines 578–584, p. 25; and lines 640–644, p. 28).

Comment 2:

Addressing the initial missteps in the nuclear accident response is crucial, yet the dissatisfaction with Fukushima prefectural government and Fukushima medical university is entirely unmentioned. This raises questions about the survey's bias (selecting issues that are easier to discuss with the respondent; a cynical view might interpret this as the authors using the respondent to voice their own dissatisfaction with the national government). Therefore, a careful, fact-based approach is essential, and the questions raised by municipal government officials must also be addressed.

Answer 2:

The reviewer’s concern regarding a “cynical view” is reasonable. However, as described in the Methods section (lines 147–152, p. 7), the interviews were conducted in an open format that invited participants to speak broadly about the difficulties they encountered during the decontamination project. Rather than the interviewer steering the conversation toward national government issues, the interviewees themselves spontaneously emphasised this topic as they disclosed their experiences. Therefore, we can report that the researchers did not “prompt” or “speak through” the interviewees in any intentional manner. To be candid, the researchers were also surprised by the extent to which the MGOs articulated dense narratives concerning their strained relationships with the national government.

That said, the above explanation alone may be insufficient to address Reviewer #2’s concern regarding a potential “survey’s bias.” In particular, the absence of references to Fukushima Prefecture or Fukushima Medical University warrants further clarification. This lack of mention may reflect the possibility that, within the open-ended interview format, once interviewees began discussing their difficult relationship with the national government, both interviewer and interviewee—without explicit intention—allowed the topic to become anchored in expressions of dissatisfaction or doubt about the national government. Importantly, the hardships interviewees experienced in the decontamination project are not necessarily limited to their relationship with the national government; we explicitly state this as the fourth limitation (lines 653–661, pp. 28–29).

The fact that no references were made to Fukushima Prefecture or Fukushima Medical University does not necessarily indicate that interviewees have no dissatisfaction toward these entities. At the same time, we believe that the accounts concerning MGOs’ strained relationship with the national government are valuable in their own right and deserve to be reported independently of a more comprehensive investigation that might also include dissatisfaction toward Fukushima Prefecture or Fukushima Medical University. Based on these considerations, we articulated our position in the Limitation section.

Comment 3:

I welcome sharing my comments with those who assisted with the survey and other staff members. I recommend obtaining approval from each municipality at least for the final draft to be submitted.

Answer 3:

Thank you for your suggestion. We discussed this issue within our research team and carefully reviewed our procedures and positionality.

The reviewer’s advice that approval from the municipalities would have been preferable is indeed important. However, some of the interviewees had already retired from their respective municipalities and were no longer under municipal authority. Moreover, we took great care to protect confidentiality by withholding not only interviewees’ names but also the names of their municipalities. In light of these circumstances, we determined that obtaining municipal approval was not required and that the study did not violate ethical standards for medical research or the journal's ethical guidelines.

******************************************************

Q2. Title

A) If authors believe there are limitations to generalization, why not explicitly state that it is a case study?

Answer for A): Although the findings of qualitative research are grounded in specific cases, they can be transferable to other periods and social contexts; therefore, we do not consider this study to fall under a conventional “case study.” We have clarified this line of reasoning in lines 620–627 on p. 27 and lines 680–689 on pp. 29–30. Additionally, for practical reasons, this journal does not accept case studies, so “case study” was not an available submission category.

B) It states that “radiation decontamination,” but what was decontaminated was not radiation but radioactive material; the term “radiation decontamination” is not rationale.

Answer for B) : The reviewer’s point is valid. We have revised the relevant expressions in both the title and the main text; for example, we changed the term to “the decontamination project of radioactive materials.”

Q3. Abstract

A) Line 35: “may be misaligned with local needs;”

B) Line 39: “limited human and financial resources”

Answer for A-B): As noted at the beginning of this response document, these expressions have been removed from the abstract as a result of our fundamental revision of the analysis.

4. Introduction

A) Line 49: “play key roles”

Citizens are also key contributors to decontamination efforts in their communities, particularly when establishing temporary storage sites. In Date City, which led decontamination activities early on, progress was made through collaboration between citizens and municipal governmental officials. This initiative is outstanding and offers tremendous value for learning. However, city employees who provided favors to researchers have faced disciplinary action. Citizens who denounced this outcome remain entirely unconvinced. Understanding these complex circumstances should be a fundamental prerequisite.

Answer for A): We agree with the reviewer that citizens played an important role. However, with respect, the fact that citizens were indeed significant actors does not contradict the present study’s focus on municipal government officers (MGOs). In the decontamination project, not only citizens but also politicians, specialists, and many other actors contributed in meaningful ways; in fact, the number of potentially relevant actors is virtually unlimited. Accordingly, it would be unreasonable to attempt to address all of them within a single manuscript. In an academic paper, it is necessary to maintain a focused discussion centred on the primary group of interest. Since a valid and coherent analysis can be conducted of MGOs—and specifically of their relationship with the national government—without discussing citizens, we believe that revisions in this regard are not required.

B) Line 60: “mandated by the national government”

Is this an appropriate relationship between the national government and municipal governments? Hasn't it become necessary to respond to residents' needs?

Answer for B): Discussing whether the relationship between the national government and municipal governments is appropriate would be redundant in the Introduction. Therefore, we have decided to retain the current wording.

C) Line 65: “little is known”

It is advisable to derive research hypotheses based on prior studies and statistical data, incorporating existing knowledge. The authors also have access to information referencing document number 41. In addition to the study listed below, surveys were also conducted by the Fukushima Prefectural and Municipal Workers Union.

<https://doi.org/10.11361/reportscpij.21.4_439>

Answer for C): Thank you very much for introducing these valuable references. Because this study focuses on MGOs’ burdens within the context of the relationship between the national and municipal governments, we believe that the current level of detail regarding MGOs’ general situation and challenges is sufficient for the logical development of the manuscript. Therefore, we have retained the existing description.

D) Line 77: “These characteristics may be the reasons for the reluctance of the affected MGOs to disclose their views regarding the national government”

It is thought that a considerable number of local government officials are involved in this work. Even with anonymity guaranteed, was it considered difficult to secure their cooperation for the survey? I believe there have been a certain number of instances, at least within Japan, where local government employees have presented their views to public.

Answer for D): We appreciate the reviewer’s important comments regarding research design and methodology. Even with full anonymity, we believe that a survey method would have made it extremely difficult to investigate MGOs’ subjective distress in the context of their difficult relationship with the national government, which is the focus of this study. For instance, if we were to imagine conducting a questionnaire survey, the researchers would need to ask items such as, “Do you have any dissatisfaction with the national government? If so, what are they?” Such items would clearly alarm respondents (MGOs), and municipalities would likely not approve a survey containing such questions.

In contrast, this study conducted in-depth interviews with MGOs with whom the researchers had established a strong rapport, enabling inductive data collection by inviting participants to speak broadly about their difficulties with the decontamination project without restricting them to issues involving the national government. This approach allowed us to avoid arbitrary topic selection and represents a clear methodological advantage of our qualitative design—one that would be difficult, if not impossible, to replicate through a survey.

As the reviewer notes, local government employees may, in some cases, express their views publicly. However, to the best of our knowledge, there are no prior academic studies in which MGOs directly involved in the decontamination project have articulated their experiences regarding their relationship with the national government in concrete terms. For this reason, we believe the necessity of our study is evident.

E) Line 79: “their anonymity must be protected;”

Respect for the autonomy of the parties involved is necessary.

Answer for E): We likewise recognise the importance of respecting autonomy. However, the key issue in this context is avoiding the identification of respondents—that is, maintaining anonymity. Introducing a discussion of autonomy here risks complicating the argument and reducing the clarity of the manuscript. Therefore, we have decided to retain the current wording.

F) Line 85: “in a difficult relationship with the national government.”

What are the administrative science-based reasons for the difficulty?

Answer for F): The relevant sentence has been removed from the current revision.

G) Line 88: “potential countermeasures”

Given the amount of time that has passed, authors should be able to make proposals based on scientific evidence that incorporates all previous attempts.

Answer for G): The relevant sentence has been removed from the current revision. Because the study now adopts the following revised objective, “potential countermeasures” is no longer included (lines 87–89, p. 4):

“The purpose of this study is (1) to descriptively understand the nature and causes of subjective distress among MGOs and (2) to make a hypothetical model of the process through which such subjective distress becomes manifest.”

H) Line 89: “hypothetical model”

Readers are interested in the theoretical basis for setting hypotheses and how those hypotheses were verified.

Answer for H): With respect, the reviewer’s reasoning is not fully applicable to qualitative research. Qualitative studies are not designed to test hypotheses; rather, they are conducted to generate hypotheses. Moreover, the hypotheses produced through qualitative inquiry do not take the form commonly seen in quantitative research—for example, “A influences B.” Instead, they describe underlying structures or coherent patterns observed within individuals or groups.

The hypothetical model presented as the outcome of this study is an appropriately derived representation of MGOs’ experiences, generated by integrating interviewees’ narratives. It is important to note that the term “hypothetical” may also be used for conceptual products that emerge at the final stage of interpretation and theorisation.

Equally importantly, our hypothetical model remains open to future examination and refinement by subsequent researchers. We hope that the present findings will serve as a starting point for further research aimed at supporting MGOs.

5. Methods

A) Line 104

Is the presentation of gender and attributes in accordance with the subject's wishes?

Answer for A): The manuscript content was reviewed and approved in advance by the study participants; explanations and materials were provided in their native language rather than English.

B) Line 243: “Line 104”

It is self-evident that even a very limited interview with a single subject can yield meaningful insights. Furthermore, how can the insufficient recruitment of subjects be explained in light of this study's objectives? Incidentally, is the main analysis in this paper supported by the subjects of this research?

Answer for B): As explained in the “Data Collection” and “Saturation Assessment” subsections of the Methods section, the sample size and the depth of the data need to be understood within the frameworks of qualitative research. Within these established frameworks, we consider the recruitment strategy employed in this study to be appropriate. Therefore, with respect, the reviewer’s comment regarding “insufficient recruitment of subjects” is not applicable.

Furthermore, the analytical findings were reviewed in advance by the study participants, who confirmed that the interpretations did not differ from their original intentions.

6. Results

Answer: Although the reviewer kindly provided comments A–H, as noted at the beginning of this response, the Results section has been substantially revised following our re-analysis. Therefore, we ask for your understanding in omitting point-by-point responses to the previous comments on the Results section. We sincerely apologise for the additional burden this may place on the reviewer, and we would be grateful if you could kindly review the revised manuscript and share your further comments.

7. Discussions

A) Line 541:

It would be better to provide specific examples of instances where government agencies provided information that was not “a realistic and contextually grounded alternative.”

Answer for A): We would like to clarify that there may be a misunderstanding. The sentence in question does not refer to government agencies; rather, it explains that our proposed improvements are grounded in MGOs’ working conditions and burdens. Therefore, the current wording remains appropriate and coherent.

8. Conclusions

A) Line 19, Page 16: “The of ages of artifacts…”

The first “of” would be unnecessary.

Answer for A): The sentence in question has been removed from the current revision.

B) In the conclusion, authors should concisely state what this study has revealed. From this perspective, the introduction must state what research hypothesis this study aims to clarify and why verifying that hypothesis is important.

Answer for B): Please note that, as part of the current revision, the first paragraph of the Conclusion section has been completely updated.

Importantly, the reviewer’s reasoning holds even without the establishment of a formal hypothesis. In qualitative research, the necessity and purpose of the study are articulated not through a predefined hypothesis but through an emphasis on a research question—one that is not known at the outset and is intended to be explored. Within this qualitative logic, the present study states its objective in the Introduction as follows (lines 87–89, p. 4):

“The purpose of this study is (1) to descriptively understand the nature and causes of subjective distress among MGOs and (2) to make a hypothetical model of the process through which such subjective distress becomes manifest.”

Please confirm that the conclusions are consistent with the objective stated above. These elements constitute a legitimate and coherent linkage between the Introduction and the Conclusion.

8. Data availability statement

While it is considered to be anonymized, it would be beneficial to express opinions on the disclosure of speech records from the perspective of open science.

Answer: Thank you very much for your thoughtful comment. We reviewed the journal’s guidelines, consulted with the editor, and considered an appropriate Data Availability Statement.

9. Table 2

Answer: As part of the current revision, Table 2 has been replaced with a list of theories generated from the interviewees’ narratives, and the staged model has been moved to Table 3. In the Results section of the main text, we extracted the necessary narrative elements to enhance clarity and improve the interpretation. Because these revisions were extensive, it has become difficult to provide point-by-point responses to each comment. We would be grateful if you could kindly conduct a renewed review of the revised manuscript. If any points remain unclear thereafter, we would sincerely appreciate your further guidance.
