# Supplementary material for: Municipal officials’ subjective distress in coordinating with the national government during the decontamination project of radioactive materials in Fukushima: A qualitative study
Source: PLoS One. 2026 Apr 3;21(4):e0346597. doi: 10.1371/journal.pone.0346597 (PMC13048426; doi:10.1371/journal.pone.0346597)
Supplement: Attachment — Submitted filename: response_R2_2.docx [file pone.0346597.s005.docx]

We wish to express our appreciation to the reviewers for their insightful comments on our paper. The comments have helped us significantly improve the paper.

Here, we put our point-to-point responses to the reviewer’s comment.

The revised texts were marked by yellow in the manuscript.

[Reviewer 2]

1. General comments

> Of course, a single paper cannot resolve all issues concerning overly complex challenges as > authors explained, but isn't the framing of the problem important?... Given that the authors

> have built relationships with research collaborators over many years, they should highlight

> the benefits this has brought to local government workers. Authors may share this reviewer's

> comments with the local government.”

Answer:

We sincerely thank the reviewer for these thoughtful and encouraging comments regarding the value of qualitative research and the broader challenges surrounding relationships between municipal and national government officials. We agree that improving intergovernmental relations is a critical step toward addressing fundamental challenges in post-disaster reconstruction.

At the same time, the present study was framed to focus on the perspectives and experiences of municipal government officials (MGOs) to illuminate an underexplored experiential dimension of their engagement in coordinating with the national government during a decontamination project. While future research incorporating the perspectives of national government officials would be valuable, this was beyond the scope of the current study. We appreciate the reviewer’s reflections and take them as an important reminder of the broader institutional context in which our findings are situated.

In addition, although this lies beyond the scope of the present manuscript, our broader research project is ongoing, and we are preparing to share relevant findings with local governments. Through such feedback, we hope to enhance the practical and developmental value of our work and, modestly, contribute to both post-disaster reconstruction efforts in Fukushima and the health and well-being of the MGOs involved.

Q1:

> A) Line 49: “play key roles”

> The reviewer believes that the burden on city employees regarding decontamination

> projects stems from their relationship with residents, but t reviewer respects the authors'

> choice not to agree with this (primary group of interest in an academic paper should be

> focused). However, the reviewer has doubts about the social nature of the argument

> presented in this paper (whether it conveniently focuses only on easily discussable

> elements).

Answer:

Thank you for this thoughtful comment. We acknowledge the reviewer’s view that the burden experienced by MGOs in decontamination projects is closely tied to their relationships with residents, and we agree that this dimension is socially important.

At the same time, as the reviewer notes, the present study intentionally focused on a specific relational domain—namely, the relationship between MGOs and the national government—in order to examine an underexplored aspect of policy implementation from the perspective of MGOs. This focus does not imply that other relationships, including those with residents, are less significant. Rather, our analytical choice reflects the need to delimit the primary group and relational context of interest in an academic study.

We also take seriously the reviewer’s concern regarding the social nature of the argument and the possibility that the analysis may privilege more easily discussable elements. In response, we have strengthened the Limitations section to explicitly acknowledge that the experiences reported in this study represent only part of MGOs’ broader social experiences and may not capture their entirety (underlined/marked, lines: 676-678, p.29):

*“Fourth, it should be acknowledged that the difficulties disclosed by the interviewees—such as their subjective distress or their strained relationships with the national government—represent only part of MGOs’ experiences and may not capture them in their entirety. It is possible that interviewees found it easier to speak about their difficult relationships with the government; if so, we must consider the possibility that some information remains undisclosed. MGOs’ experiences are embedded in a broader set of social relationships—including those with residents, colleagues, and other relevant actors—which were not fully examined in the present analysis. There is an appropriate time to elicit and understand MGOs’ experiences. In this study, interviewees were able to share their stories because a long period had passed since the disaster, and conditions were in place to ensure that no individuals could be identified. However, building a longer-term relationship of trust between researchers and participants may create further opportunities for a richer and more in-depth qualitative inquiry.”*

Q2:

> B) Line 60: “mandated by the national government”

> The reviewer respects the authors' judgment that discussing this

> point is redundant, but considers it factually incorrect in the field

> of public administration from a legal perspective as well. Note that

> the reviewer does not state that this discussion should be conducted.

Answer:

Thank you for this important clarification. We agree that the phrase “mandated by the national government” may be legally imprecise from the perspective of public administration. We have therefore revised the wording to avoid this implication and to reflect the institutional context more accurately (lines 62-63, p.3):

*“such as risk communication with residents under national policies and guidelines”*

Q3:

> C) Line 65: “little is known”

> The validity of the analytical approach itself, which focuses

> specifically on the perspective of relationships with national

> government officials, is being questioned. Regarding the

> examination of the burden on local government employees, data

> on resignations and leaves of absence among young employees

> in each municipality may also be available.

Answer:

Thank you for this important comment. We agree that administrative data, such as resignation and leave-of-absence records, may serve as useful indicators of occupational burden among MGOs. However, such data do not capture how MGOs subjectively experienced and interpreted their distress, nor how specific aspects of intergovernmental interactions were perceived and meaningfully linked to their emotional burden.

To address this point, we have revised the manuscript to clarify that our study does not claim a lack of knowledge regarding the presence of occupational burden per se, but rather addresses a gap in understanding the subjective and relational dimensions of MGOs’ experiences, particularly in relation to interactions with national government policies and officials. The relevant passages have been revised accordingly (underlined/marked, lines 68-73, p.3-4):

*“However, little is known about how MGOs subjectively experienced and interpreted such strained relationships in the context of post-FDNPP accident reconstruction efforts. In particular, it remains unclear which specific aspects of national government policies or officials’ behaviour MGOs perceived as contributing to their subjective distress. While administrative indicators such as resignations or leaves of absence may signal occupational strain, they do not illuminate how that distress was experienced or meaningfully linked to intergovernmental interactions. An in-depth examination of this intergovernmental interface from the viewpoint of MGOs is therefore essential for understanding how policy implementation processes shape occupational experiences, institutional trust, and barriers to reconstruction following a nuclear disaster.”*

Q4:

> D) Line 77: “These characteristics may be the reasons for the

> reluctance of the affected MGOs to disclose their views regarding

> the national government”

> Is the authors' inference that local governments would not have

> agreed to the survey otherwise shared with the municipalities?

> Were deceptive techniques used in requesting cooperation for

> the survey from local governments?

Answer:

Thank you for raising this important ethical point. We want to clarify that this study was not conducted through municipalities as organisations, nor did it involve deceptive practices in requesting cooperation. As shown in the Methods section, two of the authors were engaged with the municipality through their professional roles in providing health support to MGOs, and the municipality was aware that research activities could be conducted as an extension of this work.

Within this context, participation in the study was sought from MGOs on an individual basis, and all interviews were conducted with the participants’ voluntary agreement and informed consent. No institutional pressure was applied, and participation did not depend on formal organisational authorisation by the municipality.

Q5:

> E) Line 79: “their anonymity must be protected;”

> The reviewer emphasizes the importance of respecting the

> autonomy of MGOs who contribute to the research. However, do

> the authors' concerns that this approach could potentially cause

> problems align with the perspectives of the MGOs themselves?

Answer:

Thank you for this thoughtful comment. We want to clarify that the approach to protecting anonymity and the timing of publication were closely aligned with the perspectives and decisions of the MGOs themselves, rather than being based solely on the authors’ concerns.

At the time of the interviews, some participants explicitly expressed that the public disclosure of certain narratives would not be appropriate at that stage, whereas they were willing to share their experiences. The authors maintained ongoing professional relationships with the MGOs after the interviews, and the decision to publish the narratives was revisited through discussion with the MGOs over time. Ultimately, publication proceeded only after the MGOs themselves judged that disclosure was appropriate and gave their consent.

In this way, the protection of anonymity and the delayed publication were implemented as part of a process that respected and upheld the participants’ autonomy. We have revised the manuscript to clarify this point (underlined/marked, lines 85-90, p.4):

*“Therefore, when publishing the narratives of MGOs, their anonymity must be protected.* *In some cases, it is recognised that immediate public disclosure of such narratives may be inappropriate. Accordingly, publication may be delayed until the MGOs themselves judge that disclosure is acceptable, such as after transfers or retirements that reduce the risk of identification. Through such publication strategies, both the protection of MGOs’ anonymity and respect for their autonomy in research participation can be ensured.”*

Q6:

> H) Line 89: “hypothetical model”

> Readers are interested in general hypotheses and how those

> hypotheses/research questions were verified. Can the insights

> gained be applied to improve operations on the ground?

Answer:

Thank you for this comment. We agree that readers are interested in the broader implications of the model. We want to clarify that the model developed in this study is exploratory and hypothesis-generating, rather than intended for hypothesis testing.

In response to the reviewer’s suggestion, we have revised the manuscript to clarify the model’s conceptual positioning and expanded the discussion to address how the model’s insights may inform practical improvements in on-the-ground operations, including support for MGOs and intergovernmental coordination processes.

At first, the authors have changed the term “hypothetical model” to “exploratory conceptual model” to clarify the hypothesis-generating nature of our study throughout the manuscript.

Second, we have added the descriptions regarding the role of our model to the first paragraph of “Exploratory conceptual model of MGOs’ subjective distress: Reorganising theories” subsection in the Discussion section (underlined/marked, lines 537-541, p.23):

*“Here, we present the exploratory conceptual model and explain it. Note that this conceptual model developed in this study does not aim to test predefined hypotheses but to illuminate key points at which subjective distress emerges in MGOs’ work processes.”*

Third, we have revised our manuscript to explicitly distinguish the explanation of our model from its application/implications for the ground. In “Exploratory conceptual model of MGOs’ subjective distress: Reorganising theories” subsection in the Discussion section, the following revisions have been added:

*“...**as well as those between national government officials and MGOs. Our model suggests that in the early phase of the post-radiation-disaster decontamination project, improvements in inter-ministerial communication, as well as communication between ministries and municipal governments, would likely play an important role in alleviating the mental burden on MGOs.” (underlined/marked, lines 570, p.25)*

*“**Our model may be applicable for the support of the MGOs who were in a dilemma. While such individual-level mental health interventions are indispensable for supporting MGOs, it is important to recognise the risk that organisations may overly attribute the dilemma to “individual adaptability,” thereby overlooking conflicts rooted in structural deficiencies or flawed policy design...” (underlined/marked, lines 591, p.26)*

*“In Stage 3, the loss of residents’ trust in MGOs—and the resulting decline in morale and motivation—is described. Even when MGOs strive to fulfil their responsibilities while navigating the role conflict outlined in Stage 2, such efforts do not necessarily lead to positive outcomes; instead, they may result in a weakening of the ties between MGOs and residents. Our model suggests the need for an approach to improve morale and motivation among MGOs, which are influenced by exacerbated relationships with residents. From the perspective of public service motivation theory [29]...” (underlined/marked, lines 603-605, p. 26)*

*“Stage 4 represents the final phase, in which MGOs experience a sense of 'emptiness' as the core manifestation of their subjective distress. The implication of our model, which suggests that a sense of emptiness may be positioned at the final stage of the process of subjective distress among MGOs, is the importance of equipping mental health support with a diverse range of approaches to address this sense of emptiness. Because clinical outcomes of emptiness include suicide [35], the importance of addressing such emptiness is evident...” (underlined/marked, lines 624-627, p. 27)*

Q7:

> 5. Methods

> A) How did authors explain the necessity of indicating gender?

Answer:

Thank you for this question. The indication of gender was not intended for comparative or gender-based analysis, but rather to provide contextual information that allows readers to assess the interpretability of the illustrative narratives, in line with the principles of thick description in qualitative research.

In the municipality where the participants worked, all MGOs who were directly involved in negotiations and coordination with the national government were male at the time. As such, indicating gender does not increase the risk of identification and does not conflict with our approach to protecting anonymity. We have clarified this rationale in the “Study design and recruitment of participants” subsection in the Methods section (underlined/marked, lines 120-124, p.6):

*“local residents after the decontamination work started...Note that participants’ gender was reported to provide contextual information that enables readers to assess the interpretability of the narratives, consistent with principles of thick description in qualitative research. In the study context, all MGOs involved in coordination with the national government were male, and thus reporting gender did not increase the risk of identification or compromise participant anonymity.”*

Q8:

> B) Line 243: "Line 104"

> Is the authors' judgment that this study does not constitute "insufficient recruitment" based

> on the opinion that further expansion of the investigation is unnecessary?

Answer:

Thank you for this comment. Our judgment that the sample size was appropriate does not imply that further investigation with additional participants would be unnecessary. Rather, it reflects a methodological decision grounded in the information power framework, taking into account the study’s specific aim, the case-level analytical strategy, and the depth of the interviews.

We acknowledge that the population of MGOs eligible to participate in studies like ours is highly specific and that an approach focused solely on increasing sample size has limitations in this context. At the same time, while further studies may be conducted to examine or extend the explanatory model generated in this study, we consider the present findings to have independent value as an analytically grounded, hypothesis-generating contribution.

Q9:

> 7. Discussions

> A) Line 541:

> The authors' proposed improvements represent "a realistic and contextually grounded

> alternative," which I understand to mean that the authors recognize the national

> government's approach is not "a realistic and contextually grounded alternative." Therefore,

> the reviewer's suggestion here is that explicitly stating what aspects of the national

> government's approach are not "a realistic and contextually grounded alternative" could

> help facilitate improvements at the local level.

Answer:

Thank you for this comment. We want to clarify that our use of the term “alternative” was not intended to position the proposed approach in opposition to, or as a replacement for, the national government’s policies or communication strategies.

Rather, the proposed approach is framed as an alternative to placing additional communicative and emotional burdens on already strained MGOs by exploring supplementary means of fostering trust between municipalities and residents. Our intention is to propose a realistic, contextually grounded option that complements existing institutional efforts, rather than to evaluate or critique the national government’s approach. We have revised the wording in the manuscript to clarify this point (underlined/marked, lines 619-621, p. 27):

*“**Therefore, rather than placing further communicative burdens on MGOs, the improvement strategy proposed here is intended as a realistic and contextually grounded option for supporting trust-building between municipality and residents, without adding new demands on MGOs or positioning itself as a substitute for existing national policies.”*

We would also like to add a final clarification. The authors fully recognise that the critical examination of existing national policies is an important role of academic inquiry. At the same time, the primary emphasis of the present discussion was on proposing a feasible, contextually grounded way to support trust-building at the local level without imposing additional burdens on already strained MGOs.

The revisions described above were made in line with this intention. The proposed approach should therefore be understood as a complementary option within the existing institutional landscape, instead of a claim that alternative or critical examinations of national policies are unnecessary.
